# Progress in Neurology

**Published:** 1902-09-06

**Authors:** 


### PROGRESS IN NEUROLOGY.

**Word-blindness.**—Hinshelwood[1] has published an account of four cases of word-blindness, of which the most interesting is as follows:—A man, aged 35, who had previously had a left hemiplegia, became suddenly aphasic, with no loss of consciousness or motor paralysis. The aphasia was at first complete, both motor and sensory. The condition rapidly improved, and a week later these defects were only partial; there was still some motor aphasic defect, and traces of auditory aphasia, but the word-blindness persisted in a marked degree. There was no letter-blindness, but considerable word-blindness, more marked for written than printed words. The patient knew four languages, and the remarkable fact was discovered that the word-blindness extended to only three of these, his power of reading Greek remaining intact. Of the three languages affected—Latin, French and English—the degree of word-blindness was not the same in each, being greatest with English, less with French, and least of all with Latin. There was no impairment of the power of reading musical notes or numbers. The history strongly suggested syphilitic arterial disease, and rapid improvement occurred under the use of mercury and iodides. Such a case supports the view that within the visual-memory centre visual images are separately grouped. If the whole centre be destroyed, the individual is word-blind to all languages; but if there be only a partial interference with the centre, the word-blindness may be limited to a single group of these visual images,

*i.e.*, to a single language. Similarly, in the auditory memory, the different languages are arranged in separate groups; thus a teacher of French became word-deaf to French, his native tongue, but could understand what was said to him in English. Such cases have been only seldom recorded, probably from lack of testing the patient's powers, it being generally assumed that if a patient is word-blind or word-deaf to one language he is so to all.

**Meningitis.**—Sweet [2] has published an account of six cases of meningitis, with two deaths, occurring in a house within a period of two months. In three cases, those of the father, mother, and aunt, the symptoms consisted of violent headache, sickness, stiffness of the neck, and constipation; and recovery took place in about four days. One boy had headache, vomiting, and grinding of the teeth at night, and gradually recovered. Two children died, one a girl, aged 6 years, developed headache, vomiting, and stiffness of the neck, and subsequently optic neuritis; she gradually became insensible and died, but at no time had convulsions, squint, irritability, photophobia, or retraction of the head. In the last case, also fatal, aged 2½ years, there was sickness and headache, and subsequently convulsions, squint, and slight retraction of the head. The cause of this house epidemic could not be discovered, but no more cases occurred in the family when removed to another house.

**Benedikt's Syndrome.**—Hawthorn and d'Astros [3] have published a case which confirms the localising value of Benedikt's syndrome—viz., a partial paralysis of the ocular muscles of one eye and paresis of the opposite side of the body, which characterise lesions of the upper half of the cerebral peduncle. A child, aged 21 months, presented oculomotor paralysis of the right eye and paresis with involuntary movements of the left upper and lower limbs. There were oscillations and rhythmic movements of the left forearm which varied in amplitude and rapidity, occurring when the patient was awake, and ceasing during sleep. The left leg lay extended and immobile with the toes semi-flexed, but the foot showed rhythmic movements of extension of flexion. Both knee-jerks were exaggerated, but no Babinski symptom was present. Post-mortem, a tuberculous tumour was found in the upper half of the cerebral peduncle, situated mainly to the right of the middle line.

**Syphilitic Spasmodic Paraplegia** has, according to Marie,[4] symptoms which are characteristic. Though the gait is spastic, there is also paralysis of the flexors of the lower limbs. Flexion of the thigh on the pelvis is performed with great difficulty owing to the weakness of the psoas and iliacus muscles, and similarly there is feebleness of flexion of the knee and of dorsal flexion of the foot. Adduction of the thigh is enfeebled when the knees are close together, probably due to paralysis of the pectineus. There is no incontinence in the ordinary sense, but an imperious desire to micturate and to defecate which must be satisfied at once. Sensory affections are rare. Both sexual desire and power are affected, and the cremaster reflex is often abolished. There may be paræsthesia or weakness of the muscles of the upper limbs. Frequently there is increased susceptibility to emotion, and a spasmodic tendency to laugh or cry. These symptoms are so constant that they constitute a well-marked type. The morbid anatomy is unknown.

1 Lancet, Feb. 8, 1902. 2 Lancet, July 19, 1902. 3 Revue Neurologique, May 15, 1902. 4 Lancet, March 15, 1902.

*(To be continued.)*

